# Do heritable immune responses extend physiological individuality?

**DOI:** 10.1007/s40656-022-00549-0

**Published:** 2022-11-24

**Authors:** Sophie Juliane Veigl

**Affiliations:** Institut für Philosophie, Universität Wien, Wien, Österreich

**Keywords:** Evolutionary individuality, Biological individual, Physiological individuality, Organism, Environment, Small RNAs

## Abstract

Immunology and its philosophy are a primary source for thinking about biological individuality. Through its discriminatory function, the immune system is believed to delineate organism and environment within one generation, thus defining the physiological individual. Based on the paradigmatic instantiations of immune systems, immune interactions and, thus, the physiological individual are believed to last only for one generation. However, in recent years, transgenerationally persisting immune responses have been reported in several phyla, but the consequences for physiological individuality have not yet been explored. In this article, I will introduce an invertebrate immune system that is RNA-based and operates through a heritable silencing/licensing paradigm. I will discuss how such a perspective on immune systems can illuminate our conceptions of individuality. I will particularly introduce an account of immunological individuality that is not restricted to one generation.

## Introduction

What is immunogenic, that is, triggers an immune system, and what is not, sits at the core of immunology and its philosophy (Burnet & Fenner, 1949; Matzinger, 1994; Pradeu, 2011). Because of their discriminatory function, immune systems necessarily touch upon individuality: Classifications of what can persist as part of the organism, what is tolerated, and what is expelled have been proposed to constitute a critical perspective on the boundaries of the individual. A distinction between “evolutionary” and “physiological” individuality has been introduced to conceptually distinguish those units subjected to natural selection forces from those that are coherent wholes only within one generation. Immunology and its philosophy have become particularly popular ways of demarcating the physiological individual, an entity that is only coherent within one generation, from its environment (Pradeu, 2016). As the immune system is generally regarded as a policing system, a system that tells apart what belongs and what does not, what should be inside the organism, and what should not, it appears as a prime choice for a system that defines the borders of the organism.

Immune responses and, thus, the physiological individual in its immunological reading, however, are generally believed not to persist transgenerationally. This presupposition is based on how cell-based immunity is realized in jawed vertebrates – jawed vertebrates’ immune responses are confined to one generation. So far, the immunological interpretation of physiological individuality has therefore been conceptualized as strictly intragenerational.

A multiplicity of interconnected reasons can explain this focus on the particularities of jawed vertebrate immune systems^1^. For once, immunology is historically and currently a highly instrumental discipline (Rosenberg, 1994). Like many other life sciences, it is focused on understanding and treating human diseases. Therefore, it emphasizes models representing human diseases, e.g., mice, mouse cell lines, and human cell lines. In addition, the realm of jawed vertebrates marks an essential distinction within the immunological discipline – separating organisms traditionally considered to display an adaptive immune system and those that do not (Müller et al., 2018).

This is, however, problematic for two reasons. On the one hand, while some argue that the discourse regarding individuality still suffers from a too restricted diet of examples (a type of Wittgensteinian idealization (Kinzel & Kusch, 2018)) – an occurrence that has been coined the “problem of the paradigm” (Haber, 2013), the literature on biological individuality is considerably more diversified when it comes to model species for hypothesizing about biological individuality, and the distinction between physiological and evolutionary individuals (and whether this distinction makes sense). So, it is odd that jawed vertebrates are still the dominant intuition pumps when considering immunological perspectives towards physiological individuality.

On the other hand, several other species have been proposed to display adaptive(-like)^2^ immunity. Amongst these candidates are mollusks, crustaceans, insects, nematodes, bacteria, and archaea (Rimer et al., 2014). Some of these adaptive(-like) immune systems have been shown to persist inter- or transgenerationally. The best-known instance is probably CRISPR-Cas9-based immunity in bacteria and archaea, inheriting nucleic acid “spacers” complementary to the genetic material of phages by incorporating these spacers in host DNA. With new instances of adaptive(-like) immune systems coming to the front, the immunological conception of physiological individuality can be assessed from fresh and diverse perspectives.

Given the rise of data on transgenerational instantiations of adaptive(-like) immunity, I contend it is necessary to re-examine physiological individually. Is it necessarily confined to one generation? And how does transgenerational physiological individuality (that is, the persistence of parental immune interactions that decide what belongs to the organism and what to the environment) – if it exists – relate to evolutionary individuality? In this article, I will introduce an invertebrate, RNA-based immune system that persists transgenerationally to discuss immunological individuality beyond jawed vertebrates. First, I will introduce small RNA inheritance in the nematode *C. elegans* as a transgenerational immune system. I show that in the nematode *C. elegans*, small RNAs occupy an essential role in negotiating the boundaries between organism and environment through a silencing/licensing system and therefore serve as an excellent case to re-examine the immunological approach towards physiological individuality. Next, I will consider the impact of the silencing/licensing system on accounts of physiological individuality and ask how transgenerational immune systems might modify our perspective on the demarcation of physiological and evolutionary individuality. Finally, I will conclude and summarize how RNA-based systems illuminate our perspective of individuality in particular and the epistemic benefits of daring an invertebrate perspective on immunological individuality in general.

## Small RNAs negotiate organism and environment in *C. elegans*

This section will introduce an empirical case study to assess its fit with the immunological conception of physiological individuality. I will first explain some basics about small RNAs in the nematode *C. elegans*. Second, I will introduce heritable small RNA responses to environmental stimuli. Third, I will introduce the capacity of small RNAs to protect germline messenger RNAs (mRNAs) from being degraded. Through its capacity of “silencing” certain transcripts and “licensing” others, I will argue that the heritable small RNA-based silencing/licensing system fulfills the discriminative characteristics of an (adaptive-like) immune system.

### Small RNAs – the basics

Small RNAs are gene-regulatory, non-coding RNAs identified in almost all known species. Studies on small RNA-related effects in the 1980 and 1990 s culminated in identifying the phenomenon of “RNA interference” (Fire et al., 1998): small RNAs interfere with, that is, change, gene expression (Veigl, 2021). Small RNAs primarily function through complementary binding of target RNAs. Mismatches, however, are sometimes tolerated (Saxena et al., 2003). Small RNAs target and often destroy complementary messenger RNAs, which inhibits the synthesis of a specific protein. Small RNAs often rely on other effector proteins such as argonautes and dicers to ensure this targeting. Both are endonucleases that cleave small RNAs (e.g., their biosynthesis) and their complementary targets (reviewed in Ketting & Cochella, 2020).

Small RNAs play important roles in defense against selfish genetic elements (Malone & Hannon, 2009), metabolic regulation (Cai et al., 2009), and defense against viruses (Hamilton & Baulcombe, 1999). They are considered immune effectors in *C. elegans* (Engelmann & Pujol, 2010). Small RNA activities have been reported in the cytoplasm and the nucleus (Castel & Martienssen, 2013). Thus, they do not only interfere with the expression of mature mRNAs but also guide argonautes to nascent mRNAs and interfere with their expression. These processes have also been shown to help amplify certain small RNA responses.

### Heritable small RNA responses to environmental triggers

In *C. elegans*, small RNAs are key players in regulating responses to environmental stimuli. One of the best-studied instances is responses to viral infections: Viral dsRNA intermediates induce the synthesis of virus-derived small interfering RNAs (viRNAs). viRNAs, in turn, guide effector molecules, such as argonautes, to viral RNAs, leading to the viral RNAs’ destruction. Thus, antiviral immunity is instantiated through sequence complementarity. Small RNAs are also involved in other responses to environmental stimuli, such as orchestrating gene regulatory changes after starvation or heat shock.

Some of these responses were shown to be heritable. RNA inheritance in *C. elegans* was first described in the early 2000s, and evidence started accumulating in and after 2006 (Vastenhouw et al., 2006). These experiments were conducted to study the transgenerational silencing of transgenes, such as, e.g., green fluorescent protein (GFP) and endogenous genes known for easily detectable phenotypes. While many silencing effects fade after the 2nd generation, targeting specific genes results in long-lasting small RNA-based silencing (Grishok et al., 2000), with some reports indicating 80 generations and counting (Vastenhouw et al., 2006). There is most consensus, however, that small RNA responses can last for at least three to five generations (Rechavi & Lev, 2017). Thus, many observed heritable small RNA responses can be labeled “transgenerational,“ meaning they persist longer than any cells in the lineage that have been exposed to the first trigger (requires three generations for self-fertilizing hermaphrodite worms and two generations for non-hermaphrodites) (Perez &Lehner, 2019).

Vis a vis these somewhat “artificial” experimental setups, more “natural” conditions were tested. Thus far, heritable small RNA responses to viruses, starvation, and heat shock have been reported (Rechavi et al., 2011; Rechavi, 2014; Ni et al., 2016). Association with specific argonaute proteins has been identified as a prerequisite for the heritability of certain small RNA species (Claycomb et al., 2009; Gu et al., 2009; Buckley et al., 2012). The example of a heritable antiviral small RNA response is the most straightforward. Given that antiviral RNAs display sequence complementarity to the RNAs of a particular virus, if they persist, they guarantee antiviral immunity in the offspring by forming double-stranded RNAs (dsRNAs) with viral RNAs upon reinfection, thus triggering the destruction of viral RNAs.

It is crucial to keep in mind that these effects are quantitative. Millions of small RNA molecules locate in each cell, competing for effector molecules. These effector molecules are necessary for small RNA-based silencing and the heritability of small RNAs. As effector molecules are limited resources, changes in small RNA concentration will change the gene-regulatory landscape. As small RNAs respond to environmental triggers, the equilibrium of types of small RNAs changes if the organism is exposed to environmental triggers. As a result, more trigger-induced small RNAs will compete with other small RNAs for effector molecules (Sarkies et al., 2013; Veigl, 2017). With changes in effector molecule occupancy, changes in small RNA-based silencing are likely to ensue.

It is thus possible to conceptually define a “small RNA state” that is the resulting equilibrium following exposure to a particular environmental trigger. Trigger-induced RNAs are heterogeneous and partially trigger-specific. The encounter of viral RNAs leads to synthesizing small RNAs complementary binding viral sequences (Rechavi et al., 2011). In addition, other infection-induced small RNAs target endogenous mRNAs in the course of infection and thus induce gene regulatory changes (Ren & Ambros, 2015). As argued above, it is the RNA state that changes; thus, e.g., decreasing concentrations of other RNAs that were not directly triggered by the environmental stimulus are necessarily part of the small-RNA response to an environmental stimulus. Given that small RNA effectors are limited resources, an increase in one small RNA species leads to, e.g., fewer small RNAs of a different species. Thus all small RNA-based responses to environmental triggers converge at the level of small RNA pool changes.

### Inheritance of germline mRNA licensing small RNAs

Recently, small RNA inheritance researchers described that the association of small RNAs with a particular argonaute protein, CSR-1, causes the expression of complementary targets (Wedeles et al., 2013). Thus, there appear to be two grand classes of small RNAs: those that silence their targets and those that license their targets or protect them from destruction. The argonaute protein HRDE-1 and the respective effector small RNAs, such as viRNAs, small RNAs silencing endogenous genes, as well as PIWI-interacting RNAs (piRNAs), small RNAs guarding the genome against transposable elements are involved in silencing processes. The argonaute protein CSR-1 and small RNAs licensing endogenous genes are involved in licensing processes. Binding to CSR-1 and HRDE-1 has been associated with activating and repressive histone modifications on complementary regions, respectively, thus engaging in feedback loops (Shirayama et al., 2012).

**Fig. 1:**
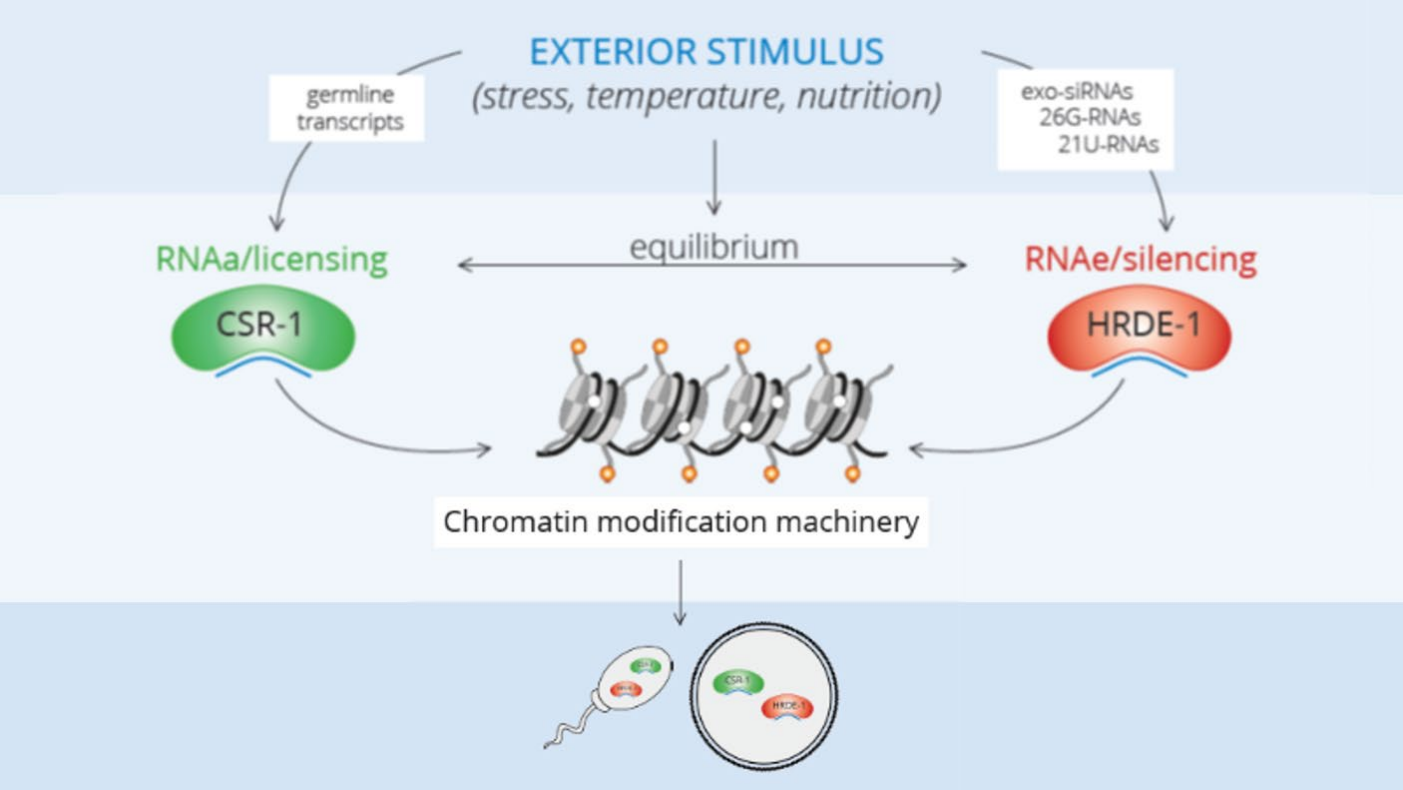
The heritable silencing/licensing state is affected by environmental triggers

Why is licensing necessary? It has been hypothesized that because worm piRNAs only require partial sequence complementarity, they could, in principle, target any mRNA (Shirayama et al., 2012; Ashe et al., 2012). Therefore, CSR-1-bound small RNAs protect those mRNAs that need to be expressed from piRNA-induced silencing. We thus have to think about the respective silencing or licensing state of an RNA as a quantitative process caused by the competition of silencing and licensing small RNAs for their complementary targets and not a switch-like decision (Fig. 1).

Does the silencing/licensing system instantiate a case of immune interactions? Immune reactions are often characterized by their intensity, instantiated through specificity, affinity, and avidity (Pradeu, 2011). This is important since it highlights the quantitative aspects of the response. I shall therefore examine these features for the silencing/licensing systems since it will further illustrate features of the small RNA state. First, specificity is realized through complementarity: a particular small RNA can only interact with a limited set of targets defined by its sequence. Second, affinity describes the biochemical interactions of two nucleic acids. Affinity increases or decreases depending on the number of mismatched base pairs and the degree of mismatch tolerance. In addition, other currently investigated processes, such as RNA modifications, could also influence affinity. Avidity finally concerns the number of binding sites. In our case, we would be talking about (depending on how long a particular target is) how many different, complementary small RNAs bind sections of the targeted RNA sequence. The more small RNAs are complementary to different sections of a target, the higher the avidity. Note the difference between the antibody and the small RNA context. Whereas in the jawed vertebrate context, polyvalency of antibodies increases the avidity, in *C. elegans*, the RNA ligand and not the receptors (the small RNAs) might be polyvalent (have more than one binding site).

This gives the idea of the “RNA state” further depth. In a particular context, effector molecules are limited resources, and environmental fluctuations will slightly alter the silencing/licensing state. For instance, an excess of exogenously supplied small RNAs, such as viRNAs, alters endogenous small RNA dynamics since they compete for the same effectors (Sarkies et al., 2013). This is in accordance with the observation that transgenes, which are usually silenced, can acquire CSR-1 targeting and thus expression (Seth et al., 2013; Ishidate et al., 2018). What the worm licenses and, thus, tolerates is negotiable. If we consider the particularities of the small RNA state with its limited effector molecules, the silencing/licensing state of a particular RNA will be shifted by environmental fluctuations if they cause changes in pools of small RNAs competing for effector binding. How organism and environments interact will also influence silencing/licensing states. The silencing/licensing system extends small RNA immune functions. Not only are there, as generally assumed, particular immune effectors that respond to environmental triggers. Small RNAs are also “inward-looking” and thus discriminate what belongs to the organism and what does not. The small RNA-based system is thus capable of performing the discriminatory functions characteristic of immune systems.

Besides displaying general immune effector functions, the silencing/licensing system also realizes immune memory. That is, repeated exposure to the same trigger, e.g., the same virus species, can be addressed faster and stronger in a specific way since small RNAs complementary to these particular viral RNAs have persisted after the first infection. Furthermore, repeated exposures have been shown to alter the silencing/licensing state of a transcript (Ishidate et al., 2018). To acquire a licensing profile over time, licensing small RNAs must, at a point, outcompete silencing small RNAs for binding to a particular transcript. Thus, at the tipping point of the equilibrium from silencing to licensing, we expect the intensity of interaction to increase for one pool of small RNAs and the intensity to decrease for another pool of small RNAs. With these changes, other aspects of the intensity could change as well. On the one hand, biochemical interactions might be different because of mismatched base pairs, mismatch tolerance, or particularities of CSR-1 vs. HRDE-1-based targeting. On the other hand, avidity, that is, the total number of small RNAs targeting particular stretches of a sequence, might decrease once the equilibrium is beyond a tipping point. Also, consecutive environmental stimuli will tip the balance towards a more intense response toward newly introduced sequences compared to a sequence that has acquired licensing. In conclusion, while interactions of ligands and receptors are always present, the intensity is expected to decrease over time for a transcript that has acquired licensing. Over time, it will be “tolerated” by the immune system.

When examining changes in the intensity of the response, we naturally have to look at downstream effects of the immune interaction, that is, interactions that are not entirely limited to RNA-RNA interactions. If tolerance means the acquisition (quantitatively) of a licensing profile, then what is left to ask is whether the overall response’s intensity decreases. It is important to note that experiments to investigate these hypotheses have not been conducted yet. It is nevertheless possible to come up with testable predictions regards the chronicity of antigen exposure in the small RNA-based system.

While there are initial amplificatory cycles after second triggers (Houri-Ze’evi et al., 2017), making second exposure to be handled faster and stronger (Sterken et al., 2014), increasing both the intensity of immune interactions through changes in avidity and the immune response, we could hypothesize that upon extended antigenic presence of, e.g., a viral RNA (that is, repeated infections, the worm living in an ecosystem with high viral presence, or an artificial system in which a transgene continuously expresses viral RNAs) might acquire CSR-1 targeting and thus triggering a modulation of the general immune response, involving non-RNA based factors. Investigating how shifts in small RNA pools might influence a general inflammatory response through, e.g., gene-regulatory changes will be a fruitful topic of investigation. These musings about the silencing/licensing system, importantly, are to be understood as a heuristics to propose or guide future investigations or to adopt a particular perspective on small RNA systems that could be beneficial epistemically but not as ontological claims about the entities and activities comprising the C. elegans immune system.

Before closing this section, it needs to be mentioned that there is much current debate on whether instantiations of immune memory, such as the small RNA-based system, should be genuinely coined “adaptive(-like).“ Settling this issue requires a separate paper. The silencing/licensing system is not functionally equivalent to jawed-vertebrate adaptive immunity, that is, for every entity and process of the jawed-vertebrate immune response, there does not exist a corresponding small RNA or small RNA effector. Also, key processes are different. For instance, while the jawed vertebrate immune response is “customized” (the immune receptor repertoire predates the first trigger), the small RNA-based immune response is “tailor-made” (complementary immune effectors are synthesized after exposure to the trigger) (Rimer et al., 2014; Veigl, 2022).

While there is no functional equivalence between the silencing/licensing system in C. elegans and B/Tcell-based adaptive immunity in jawed vertebrates, the silencing/licensing system could be considered another realization of adaptive immunity, given that it displays several important features of adaptive immunity, such as specificity, immunizability, clonality, antigen recognition, memory duration, and extinction.

What is key for the silencing/licensing system is that it suggests a particular perspective on how the organism situates itself in the environment. Every RNA is susceptible to this process. We arrive at a picture in which we look at every transcript as being constantly in the process of acquisition or being let go, sensitive to quantitative fluctuations and environmental conditions. Transcripts, over transgenerational time, blend in and out of the individual. Having introduced the silencing/licensing system, I will now explore how this particular invertebrate perspective on immune systems contributes to notions of immunological individuality.

## Transgenerational immune systems and individuality

In this section, I shall employ the silencing/licensing immune system just established to investigate the issue of immunological individuality from an invertebrate perspective. Discerning what counts as a biological individual constitutes a central problem for biologists and philosophers of biology (Godfrey-Smith, 2013; Clarke, 2013; Stencel & Proszewska, 2018). Defining a biological individual means asking what constitutes a “countable, relatively well-delineated, and cohesive unit in the living world” (Pradeu, 2016, p. 762). There are many non-coextensive conceptions of biological individuality, such as physiological, evolutionary, and developmental definitions (Suárez & Stencel, 2020). In some cases, these perspectives contradict each other regarding what counts as an individual (Smith, 2017).

Nevertheless, philosophers of biology somewhat agree that biological individuals are bound in three-dimensional space, endure, are composed of physical matter, bear properties, and participate in processes and events. Besides this minimal consensus, the question of whether there will be one comprehensive account of individuality or whether individuality can be multiply realized (Clarke, 2013) or subjected to promiscuity (Dupré, 2012) is open to discussion.

The question of individuality has traditionally been addressed from the perspective of evolutionary biology, evolutionary individuals being those upon which natural selection acts. The organism, by this account, is only one type of biological individual subjected to evolutionary forces – genes, chromosomes, cells, or populations being other possible evolutionary individuals (Maynard-Smith & Szathmary 1995; Okasha 2006; Godfrey-Smith 2009). An evolutionary individual is a unit from the point of view of natural selection. Necessary but not sufficient criteria are variation, heritability, and differential fitness (Okasha, 2006) – Darwin’s descent with modification. There is, however, debate concerning the specifications of these criteria, for instance, whether heritability must be realized vertically or whether heritability can be established exclusively through reproduction events. Other often-mentioned features are the division of labor, coordination amongst the individual’s parts, and collective interest (Hull, 1980; Buss, 1987; Godfrey-Smith, 2009; Maynard-Smith & Szatthmary, 1995; Stencel & Wloch-Salamon, 2018). Several units qualifying as evolutionary individuals have been proposed, such as the replicator/interactor (Dawkins, 1976; Hull, 1980), the reproducer (Griesemer, 2000), the Darwinian individual (Godfrey-Smith, 2009), and the reconstitutor (Veigl et al., 2022).

Individuals are, however, not necessarily entangled with selection. In recent years, defining and elaborating on individuality beyond the evolutionary individual has gained more and more traction. While certain evolutionary individuals, such as genes, or chromosomes, are not physiological individuals, not all physiological individuals can form lineages and thus be subjected to natural selection. For instance, there is a current debate on whether the holobiont – the host plus all its microorganisms - is only a physiological or also an evolutionary individual. Following Godfrey-Smith (2009), many scholars seem to agree that there is, however, a space where physiological and evolutionary individuals overlap, examples being fruit flies or the aphid *buchnera* multi-species organism.

One current approach to define physiological individuality is the immunological perspective, specifying the physiological individual in terms of its immune interactions. Pradeu lists three main reasons why the immune system should be considered a significant contributor to our notions of physiological individuality: It is discriminative, systemic, and present in all biota. “An immunological individual is a functionally integrated whole made up of heterogeneous constituents that are locally interconnected by strong biochemical interactions and controlled by systemic immune interactions.“ (Pradeu, 2016, p. 8). The boundary established by the immune system is the boundary of the organism. Thus, entities that interact with the immune system but are not eliminated are part of the physiological individual.

The small RNA-based silencing/licensing system, defining the results of immune interactions as the expression or repression of a target, introduces a new perspective on immunological individuality. In *C. elegans*, based on the silencing/licensing system, the physiological individual is confined by those RNAs that are expressed and can perform their functions (regulatory, being translatable). What counts as the individual is thus quantitative, continuously renegotiated, and responsive to environmental fluctuations. What defines the small RNA-based immune system is the capacity to establish complementarity to every possible RNA through (1) genetically encoded small RNAs; (2) mismatch tolerance; (3) RNA-dependent RNA polymerase-dependent synthesis of complementary small RNAs (that could be of exogenous origin).

One particular aspect of the silencing/licensing system is its heritability. Heritability, however, is usually not considered an aspect of the immunological individual but rather constitutes one of the minimal, necessary, but not sufficient criteria of evolutionary individuals. Given immunology’s focus on jawed vertebrates, immune interactions are generally believed not to be heritable in a stable way, even though some immune effectors are transferred through material overlaps from parent to offspring in jawed vertebrates (e.g., antibodies). These transfers are, however, believed not to extend the intergenerational as there are no dedicated processes yet uncovered that could ensure the transgenerational persistence of these effectors. We thus have to ask how much our notion of physiological individuality is based on a particular notion of immunity that, in turn, is inspired to a large degree by a particular instantiation of an immune system.

While the immunological account of the physiological individual necessarily has to apply to all individuated biota, it is strongly influenced by how the jawed vertebrate immune system negotiates the borders of the organism. If our account of the immunological/physiological individual, however, mainly rests on the fact that cell-based immunity or its effects are not heritable, we have to ask whether we should take the special case of the jawed vertebrate immune system as a determinant for all physiological individuals and, as a result, the relation of evolutionary and immunological individuality. With the jawed vertebrate immune system considered one of the major evolutionary transitions (Müller et al., 2018), a definite separation of the immunological and the evolutionary might have come with it, but this separation might not apply to other phyla.

Before exploring how a transgenerational perspective on immune systems might alter our perspective on immunological individuality, let me emphasize that whether (pools of) small RNAs are candidates of evolutionary individuals (that is, whether they are subjected to the forces of natural selection) is an open debate. Let us, however, consider the minimal criteria for evolutionary individuality: First of all, small RNA pools are heritable. Also, it seems highly likely that a particular heritable small RNA pool contributes to differential fitness. Expressing and repressing specific RNAs in particular scenarios might contribute to the organism’s fit to the environment and alleviate or overcome specific selection pressures. In addition, the silencing/licensing state leaves room for variation since we do not expect a particular environmental change, such as viral presence, or starvation, to cause an identical response in each organism in a Darwinian population. While, e.g., viral infection causes the synthesis of antiviral small RNAs, we will expect stochastic fluctuations in small RNA states on the one hand, on the other hand, fluctuations based on previous exposures, previous ways of blending organism and environment in different individuals. Given that previous exposures influence the small RNA state and thus differential fitness, there will be a higher similarity between parent and offspring than between lines of ancestry. Thus descent with modification could be ensured.

It is necessary to mention that when characterizing the persistence of environmentally induced immune interactions as inheritance phenomena, this characterization entails a Lamarckian interpretation (Veigl, 2017) of the processes involved. Not only are environmentally induced traits that are adaptations to a particular environmental condition inherited, but the silencing/licensing system also connects to a reconstructed/operationalized version of the use/disuse paradigm that Lamarckian inheritance (one might argue, by definition) requires (Veigl, 2017; Speijer, 2019; Veigl, 2019; Loison, 2021). If there is competition for which small RNAs bind to silencing or licensing argonautes, association with CSR-1 would be a “use” signal that promotes increased expression also reciprocally, preventing repressive histone marks on complementary regions. Association with HRDE-1 then entails “disuse,“ further associated with the heritable silencing of a complementary region, which results in reduced expression. This also matches the fluid, non-discrete character of how traits emerge and change in Lamarck’s musings. In conclusion, it would be possible to characterize this instance of transgenerational adaptive(-like) immunity as an evolutionary (and mechanistically Lamarckian) adaptation (Veigl, 2022).

But let me now consider the main target of this discussion – the concept of the immunological individual. If the immunological individual is defined by the small RNA state (in C. elegans), we have to understand the immunological individual as established through a particular expression/repression profile that persists. Also, of those entities that cannot be targeted through RNA-RNA interactions directly, such as proteins or cells, however, the transcripts that brought them into being interacted with the silencing/licensing system. Both within one generation as well as beyond one generation, the individual’s boundary is constantly renegotiated. Persistence and constant re-negotiation might, at first glance, lie in conflict. The small RNA perspective, however, shows how stability and change are both necessary and are mediated through the same process. To maintain what is licensed, the organism also needs environmental cues that stimulate amplificatory loops. Thus, environmental cues are needed to maintain the boundary of the individual. This also suggests a particular view regarding the relation between organism and environment. It suggests a commitment to the inseparability of organisms and environments (internal and external), conceptualizing them as *organisms-in-the-world* and not organisms in the world (Smith, 2017). It suggests thinking of organism and environment constitutively, not interactionally (ibd.).

But I have not yet addressed what it means for the physiological individual that the immune reactions of the silencing/licensing system are heritable. This first necessitates the question of what is meant by a “generation” in the context of transgenerational immune responses. Generations of organisms are most of the time defined by reproduction events, also called “the bottleneck” view: As an individual reproduces, a new generation begins through progeneration. This definition is situated, however, in the evolutionary discourse about units of selection. But with regards to individuality conferred by the silencing/silencing system, there is an incongruency: a reproduction event, an event that makes discernable parent and offspring, does not mark a decisive event for small RNA-based individuality. Placatively speaking: If pools of small RNAs are inherited, and thus, the silencing/licensing state persists, the first encounter of a virus changes the small RNA state, the silencing/licensing equilibrium, and thus, the boundaries of the organism more than a reproduction event. In other words – an encounter with a virus will change what is expressed and what is repressed more than the start of a new generation within a stable environment.

If we follow the immunological reading of physiological individuality, that is, an individual is defined by local biochemical interactions and negotiated by systemic immune interactions (Pradeu, 2016), we would consider the physiological individual maintained as long as the systemic immune interactions show continuity. In the mammalian immune system, the transition from parent to offspring marks the beginning of a new physiological individual because there is discontinuity regarding the interactions of cell-based immunity: it is generally believed that the offspring’s immune system needs to establish its adaptive immune response anew. This concerns interactions with its “internal world,“ e.g., gut microbiota, and its external world – e.g., its interactions with certain pathogens. In this scenario, the end of one organism and the beginning of a new one also mark the establishment of a new way for the organism to deal with the interior and the exterior, i.e., a new physiological organism. In the case of the small RNA example, however, immunological individuality can extend more than one generation of evolutionary individuality. But this conclusion seems quite puzzling.

We could start with addressing this puzzlement by asking why we have the intuition that the physiological individual is confined to a generation (a concept developed in an evolutionary/genetics context). Immunological individuality was formulated with cell-based immunity in mind. Even though the philosophy of immunology aims for generality, it is highly influenced by a particular way of thinking about immune systems. Insisting on the distinction between the evolutionary and the physiological (immunological) individual is caused by a jawed-vertebrate perspective on immune systems. Introducing the small RNA-based perspective opens several possibilities for consolidating individuality, evolutionary and physiological. Also, it helps to question why physiological individuality needs to be confined in the same way as evolutionary individuality (in terms of generations). With regards to these two concepts, the notion of the evolutionary individual has been quite dominant in 20th -century discourse, and the concept of the physiological individual, while aimed at separating a distinct phenomenon, might have, to a degree, been morphed after that of the evolutionary individual. In the next section, I will assess whether it is possible to separate both concepts regarding their generationality.

## The limits of physiological individuality

In the previous section, I considered the possibility of the physiological individual being maintained as long as the systemic immune interactions show continuity. I argued that in some cases, one physiological individual might start and end at different points than one evolutionary individual. It might even last longer than one evolutionary individual. But where are the limits to physiological individuality? If, for instance, a small RNA state is transgenerationally heritable – would parent and offspring constitute one single organism, as long as the offspring maintains the small RNA state of the ancestors?

This lies in conflict with several accounts of physiological individuality. Pradeu argues that the study of a physiological individual is the study of its life from conception to death (Pradeu 2013, p. 5). An organism has to be a functionally integrated whole (2016, p. 258). “Persistence” (Burnet 1962, p. 38–41) of a cohesive whole needs to be explained by individuality. In that spirit, Godfrey-Smith (2013) and Smith (2017) have characterized organisms as essential persisters. They persist in using energy to resist the forces of decay and are (contingently) things that reproduce (Godfrey-Smith, 2013, p. 25). A particular organization is a condition for persistence, which is usually ensured through differentiation and subsequent integrations (Smith, 2017, p. 6). Therefore, development is an important aspect of organismality.

Such developmental processes involve “an ongoing, dynamic, construction and reconstruction of individuals by sub-organismic, non-organismic, and other-organismic factors.” (Smith, 2017, p. 6). Another important concept is that of the graduality of organismality: “if the parts of a system have a significant amount of metabolic autonomy, and can keep themselves going somewhat independently, this reduces the degree to which the larger system counts as an organism” (Godfrey-Smith, 2013, p. 26).

Given this perspective, the essential question for the C. elegans example is whether the reconstruction (or reconstitution, Veigl et al., 2022) of the small RNA state in the next generation can be considered part of these processes. The primary problem is that C. elegans and their offspring are not (biochemically) integrated. And if we consider biochemical integration as a necessary criterion for organismality and physiological individuality, we would have to reject that physiological individuality extends one generation. Also, if we look into research practice, C. elegans and offspring with corresponding small RNA states are not considered one persisting physiological individual (Rechavi et al., 2011; Ishidate et al., 2018). However, it is necessary to point out that small RNA inheritance research is embedded within a genetics discourse, where it is the very point to identify individuation events despite descent (and thus a high degree of similarity in DNA, RNA states, etc.…).

There are, of course, also other examples of inherited immune effectors whose functions persist inter- or transgenerationally. I shall now ask whether such instances present similar issues for physiological individuality or whether they can illuminate the problem of transgenerational physiological individuality by other means. Take, for instance, the inheritance of maternal antibodies in mammals (Lemke et al., 2004). In such a case, researchers consider mother and (birthed) offspring as two – physiological and evolutionary – individuals, even though a part of the immune response persists (the problem of pregnancy and organismality is, of course, a separate issue (Grose, 2020; de la Nuño et al., 2021)). However, one might argue that this example is too dissimilar from the small RNA example since, in the maternal antibody case, the persistence in immune effector functioning is not systemic enough – it concerns the resistance to certain pathogens, but not how the organism embeds into its environment holistically.

But there is a better example. Think about the holobiont, the organism that is thought to be the totality of the host and its microbiota. Using the example of the holobiont, Suárez and Stencel (2020) problematize the persistence perspective introduced by Godfrey-Smith and Smith because it “presupposes that functional dependency and functional integration are necessarily symmetric relations among all the parts of the ensemble” (2020, p. 10) and because it “assumes that the criteria for finding ‘cohesive, well-delineated units’ is exclusively whole-dependent” (2020, p. 11). Whole-dependence assumes that:

“A whole is a biological individual if and only if all the elements that constitute the whole satisfy a specific criterion of individuality (physiological, immunological, evolutionary, etc.).” (2020, p. 2).

Suárez and Stencel, on the other hand, support part-dependence:

“a whole is a biological individual only if all the elements that are included in the whole fulfil a certain criterion in relation to a given element of the whole that one is prioritizing.” (ibd.)

Thus, the ontological status of the holobiont differs depending on the perspective, even if the same concept of individuality is used. Suárez and Stencel explain this partly through a difference in scale between host and microbe: “the physiological necessities of the former are radically different from the physiological necessities of the latter” (2020, p. 9). From the microbe’s perspective, they claim that the holobiont is not a biological individual but an ecological community. From the perspective of the host, the holobiont is a biological individual.

But part-dependence also has a second consequence: if we take the perspective of the host’s microbiota, there are many lineages of evolutionary individuals throughout the host’s lifetime. However, they constitute one physiological individual, the host and its microbiota, the functionally integrated holobiont. In this case, the holobiont’s physiological individuality lasts longer than several evolutionary individuals of microbiota. But what about heritability? There is currently a debate about whether the holobiont is also an evolutionary individual (Bourrat & Griffiths, 2018). While there are some clear instances where microbiota are transmitted (Margulis, 1990), thus qualifying the holobiont for an evolutionary individual as well, others have argued that it is sufficient that the composition of microbiota persists for the holobiont to count as an evolutionary individual (Suárez, 2020). However, if the holobiont can persist, there would have to be a persistence (by whatever means) of immune interactions between the host and the microbiota to guarantee symbiosis between the host and microbiota. If these immune interactions persist for more than one generation, would that be a case for physiological individuality outlasting evolutionary individuality? Also, I believe, in this case, one could argue that the immune interactions of the host are not limited to interactions with its microbiota. Therefore, given other stimuli, such as exogenous pathogens, or endogenous processes that need to be addressed to guarantee homeorhesis, this example is also not one where the system that guarantees physiological individuality persists.

The example of the small RNA state seems more pervasive since it is reflective of all immune interactions: it is turned to the inside and the outside. So, do we have to grant that parent and offspring with identical small RNA states must be considered one physiological individual? And, if inheritance continues for many generations, would all descendants be considered the same physiological individual? These cases ring unintuitive to most notions we have about what an individual is.

I believe that cases of small RNAs being heritable for a long time or indefinably do not present a problem since the small RNA-based system is a system that presupposes environmental triggers so it can negotiate what is environmental and what is organismic. For two C. elegans to be considered one physiological individual in regions geographically distinct, say, after 5000 generations, we would have to assume that the environments are stable (diachronically in one place and synchronically in all places). As this condition does not hold empirically, and the small RNA state is responsive to environments, it will thus not remain the same for an extended amount of generations.

But the heritability of a small RNA state only for one generation already presents problems. C. elegans has many offspring. Worms can lay up to 140 eggs per day (and live for around 16 days) (Muschiol et al., 2009). Would all offspring from the same parent, if the small RNA state persists and the environment be somewhat stable, be considered one physiological individual? This question is reminiscent of the work of Daniel Janzen’s “What are Dandelions and Aphids?” which has occupied the philosophical literature on individuality and organismality. Through apomixis – a form of non-interspersed parts of the Dandelion – the Dandelion clones - are one evolutionary individual (1977, p. 587). Janzen makes a similar argument about aphids that grow by parthenogenesis and thus spread over the habitat.

Note that Janzen made this argument about evolutionary individuality and the unit of selection, which is not the question of this article. Nevertheless, it touches on core problems when it comes to the desiderata for a good concept of individuality which has to be successful in counting offspring (Sober, 1984; Clarke, 2010), distinguish growth from reproduction (Herron et al., 2013), and to distinguish organisms from parts of organisms (Herron et al., 2013). Still, Janzen’s example is instructive since he seems to argue that connection or integration of the parts of an individual is not important in order to count as an individual. Thus, for immunological individuality, we might also have to ask: is integration important? If yes, if it is a necessary criterion for physiological individuality, then parent and offspring with the same small RNA state do not constitute one physiological individual.

If we do not, however, take the intuition about the importance of integration for granted, another, perspectivist answer is possible: From the perspective of immunological individuality, parent and offspring with the same small RNA state constitute a physiological individual. From other perspectives on physiological individuality, such as developmental or metabolic, they do not. This is still quite an inflationary perspective on individuality. But the part- vs. whole-dependent approach can create greater clarity here by differentiating further “perspectives” when employing the immunological account. The case is, however, different from the holobiont example since we do not have the problem of “scales.” C. elegans parent and offspring are roughly at the same scales, so it makes sense to consider all C. elegans with the same small-RNA state as parts. From the perspective of the parts, each is a physiological individual since they are integrated, developed, and differentiated entities.

What would, however, a perspective of the whole, then, be? I believe it would have to be an *organism-in-the-world* perspective that considers organisms as constitutively embedded in their world: “the persisting region of biological matter about which one is concerned is always embedded in a context as it goes along living” (Smith, 2017, p. 8). While epistemically useful, a distinction between organism and environment is conceptually misleading (ibd.). Thus, from an eco-immunological perspective that shifts its focus to how organism negotiates with the environment, it is possible to grant that parent and offspring, if small RNA states persist, are one physiological individual that can extend a generation and thus can extend one evolutionary individual. This holds as long as interactions of the small RNA state in parent and offspring with the environment are stable, as long as organism and environment blend in the same way and are not disturbed through internal or external fluctuations.

## Conclusion

I commenced this article by arguing that concepts and theories formulated within immunology and its philosophy often rest on a jawed-vertebrate perspective. By introducing an alternative instantiation of an immune system, the silencing/licensing system, I tried to show that the generally upheld idea that a physiological individual is confined to a particular generation is grounded in immune responses not being heritable in jawed vertebrate immune systems as well as potentially the influence of the (older, more established) notion of evolutionary individuality on formulations of physiological individuality. Discussing the small RNA-based silencing/licensing system, I tried to show that physiological/immunological individuality is not necessarily confined to one generation and thus also provides a further potential instantiation where the physiological and the evolutionary meet.

I hope to have shown that the silencing/licensing system provides an insightful perspective on how the organismal and the environmental relate. Furthermore, it provides a mechanistic basis for how the environment can become organismal and how the organism can become environment. Finally, through its focus on the quantitativeness (of small RNA equilibria), it instantiates a perspective on the boundaries of the organism as being eternally fluid and morphed by its environment. With that perspective in hand, I tried to show that while there are approaches towards physiological individuality – developmental, organismic, part-dependent – that dissuade looking at the physiological individual as persisting inter- or transgenerationally, if the small RNA state remains the same, from a particular perspective, which is eco-immunological and organism-in-the-world focused, it makes sense to doubt that physiological individuals necessarily have to begin at progeneration events.

It is important to note that heritable small RNA-based responses to environmental stimuli and the silencing/licensing system, in particular, have been discussed in the literature exclusively in a space that is disciplinarily separated from immunology. They are considered genetic phenomena, are endowed with geneticist terminology, and are mobilized to discuss theories of inheritance and evolution in some cases. While RNA interference in a very general way, is registered as performing immune functions, both in the biological and the philosophical literature, its heritability is not part of these accounts. Heritability of immune effectors is generally not assumed to characterize immune responses. What about a particular phenomenon is included in discussions on immunology and what is neglected thus also seems partly to be caused by the dominant jawed-vertebrate perspective.

While such “problems of the paradigm” might never be entirely evaded, there are remedies to keep them in check in the philosophy of immunology. One, demonstrated here, is to question membership. It is highly likely that phenomena that could be conceptualized as immune phenomena but do not resemble the respective realization in jawed vertebrates are overlooked or left out when studying instantiations of immune systems in non-jawed vertebrate species. But it is precisely these aspects of immune responses, those that do not resemble jawed-vertebrate instantiations, we need to foreground in order to assess our perspectives on immunology and immunological individuality. We thus need to broaden our focus and expect phenomena that could be fruitfully studied as immune phenomena to be hidden within different taxa and disciplinary contexts.

For the specific case of this article, I unearthed the silencing/licensing system from the disciplinary context of genetics. I used the particularities of the *C. elegans* small RNA-based immune system to argue that our notion of physiological individuality confined to one generation is situated in a particular epistemic context. If we go beyond that particular context and admit other instantiations of immune systems to contribute to the discourses on immunological individuality in terms of how they differ from the established epistemic context, and not only in terms of what is similar, we arrive at diversified but more fine-grained accounts. Investigating other systems that negotiate (physiological) individuality will provide an even better understanding of where and how the physiological and the evolutionary meet. If the genetic and the immunological interact and coincide differently in different phyla, the same must be true for physiological end evolutionary individuals.

In conclusion, the small RNA perspective shows that the immunological/physiological individual is not necessarily confined to one generation because silencing/licensing equilibria persist transgenerationally. How should this change our perspectives on individuality? Whether separating the physiological and evolutionary individual is empirically adequate is sensitive to the particularities of the phylum in focus. And whether the physiological individual can extend the evolutionary individual might be as well. It is vital to move away from generalist statements when we have only focused on particular instantiations of the processes we are interested in. As pointed out above, we should consider the physiological and the evolutionary more or less hybrid dependent on the respective species. Also, how both relate to each other temporarily (whether one type of individuality can span shorter or longer than the other) might be species-dependent. With this article, I aim to extend this perspective and argue that physiological individuality displays different durations depending on the species. Thus, transgenerational (adaptive(-like)) immune systems are a further instantiation of an overlap between physiological and evolutionary individuality. When it comes to individuality in organisms with small RNA-based immune systems, how the organism in one generation becomes countable (identifiable as one) is causally relevant to how the organism becomes countable in the next generation. The immune system is not only adaptive in the immunological sense but might also be adaptive in the genetic, evolutionary sense. The boundary between both is fluid.

## Data Availability

On request.

## Footnotes

1 In recent years, non-jawed vertebrates have been shown to display functionally equivalent adaptive immune effectors, that evolved independently. Thus, those claims that are made about jawed vertebrate immune systems usually accommodate also non-jawed vertebrate immune systems, given them displaying B-cell-like, T-cell-like, and MHC-like effectors.

2 It is not the purpose of this paper to decide which immune systems are “truly” adaptive, and which realize another form of immune memory. For the purpose of this paper, it suffices that some discriminatory immune function persists transgenerationally. Thus, remaining undecided regard the “adaptive” character of the response, I shall use the term “adaptive(-like)” throughout this article.
